# Identification of Key Genes and Pathways associated with Endometriosis by Weighted Gene Co-expression Network Analysis

**DOI:** 10.7150/ijms.63541

**Published:** 2021-08-03

**Authors:** Jingni Wu, Xiaoling Fang, Xiaomeng Xia

**Affiliations:** 1 Department of Obstetrics and Gynecology, The Second Xiangya Hospital, Central South University, Changsha, Hunan 410011, China.; 2 Department of Obstetrics and Gynecology, David Geffen School of Medicine, University of California, Los Angeles, California 90095, United States.

**Keywords:** endometriosis, WGCNA, biological markers, pathway

## Abstract

**Background:** Endometriosis is a common gynecological disorder with high rates of infertility and pelvic pain. However, its pathogenesis and diagnostic biomarkers remain unclear. This study aimed to elucidate potential hub genes and key pathways associated with endometriosis in ectopic endometrium (EC) and eutopic endometrium (EU).

**Material and Method:** EC and EU-associated microarray datasets were obtained from the gene expression omnibus (GEO) database. Gene set enrichment analysis was performed to obtain further biological insight into the EU and EC-associated genes. Weighted gene co-expression network analysis (WGCNA) was performed to find clinically significant modules of highly-correlated genes. The hub genes that belong to both the weighted gene co-expression network and protein-protein interaction (PPI) network were identified using a Venn diagram.

**Results:** We obtained EC and EU-associated microarray datasets GSE7305 and GSE120103. Genes in the EC were mainly enriched in the immune response and immune cell trafficking, and genes in the EU were mainly enriched in stress response and steroid hormone biosynthesis. PPI networks and weighted gene co-expression networks were constructed. An EC-associated blue module and an EU-associated magenta module were identified, and their function annotations revealed that hormone receptor signaling or inflammatory microenvironments may promote EU passing through the oviducts and migrating to the ovarian surfaces, and adhesion and immune correlated genes may induce the successful ectopic implantation of the endometrium (EC). Twelve hub genes in the EC and sixteen hub genes in the EU were recognized and further validated in independent datasets.

**Conclusion:** Our study identified, for the first time, the hub genes and enrichment pathways in the EC and EU using WGCNA, which may provide a comprehensive understanding of the pathogenesis of endometriosis and have important clinical implications for the treatment and diagnosis of endometriosis.

## Introduction

Endometriosis is an estrogen-dependent gynecological disorder characterized by the growth of endometrium in ectopic locations 1. A total of 30-50% of women with endometriosis suffer from pain and/or unexplained infertility 2. Despite several theories (i.e., retrograde menstruation, coelomic metaplasia, Müllerian remnants) that have been proposed, the pathogenesis of endometriosis is still unknown. The widely accepted retrograde menstrual reflux hypothesis states that eutopic endometrium (EU) migrates and survives outside the cavity of uterus, then establishes new endometriosis lesions. It is believed that the elucidation of molecular and functional specificities of the ectopic endometrium (EC) and EU facilitates a better understanding of the complex physiopathology of endometriosis. Previous studies have shown that the EC may behave differently from its eutopic counterpart 3. However, these studies mainly focused on a few molecules or the gene expression differences between different tissues, without considering the intrinsic relationship between these genes. In addition, there is still room for improvement of the bioinformatics algorithm in analyzing these transcriptomic data, and the specific biomarkers and roles of the EC and EU in endometriosis remain uncertain. Therefore, our study for the first time explored the genomic alteration profiles of the two entities of endometriosis using weighted gene co-expression network analysis (WGCNA) to identify endometriosis-associated biomarkers and pathways.

Currently, there is no standard protocol for analyzing transcriptomic data. Network analysis is a promising direction that allows for a greater ability to recognize biological themes or pathways. It combines biology and network science to study the relationships of interacting components, which may provide novel and comprehensive insights into the diseases from the level of multiple genes 4. WGCNA is a network method for identifying highly correlated gene expression modules in different samples and analyzing the correlation between the module and disease type/clinical phenotype. Hence, WGCNA has been widely used to explore the biomarkers and therapeutic targets of various diseases, such as breast cancer 5. WGCNA has also been used to identify biologically related modules. For example, Wang et al. found fifteen hub genes that were highly correlated with the progression and prognosis of clear cell renal cell carcinoma using WGCNA 6. As a result, using WGCNA, we attempted to identify the modules of co-expressed genes highly associated with endometriosis and their key drivers. Meanwhile, we tried to explore the key pathways of the EC and EU in the pathogenesis of endometriosis. Our study may provide a better understanding of the disorder through the comparison between the eutopic and ectopic endometrium, and provide a new insight into the molecular mechanisms underlying the pathogenesis of endometriosis.

## Materials and methods

### Study design

**Figure 1:**
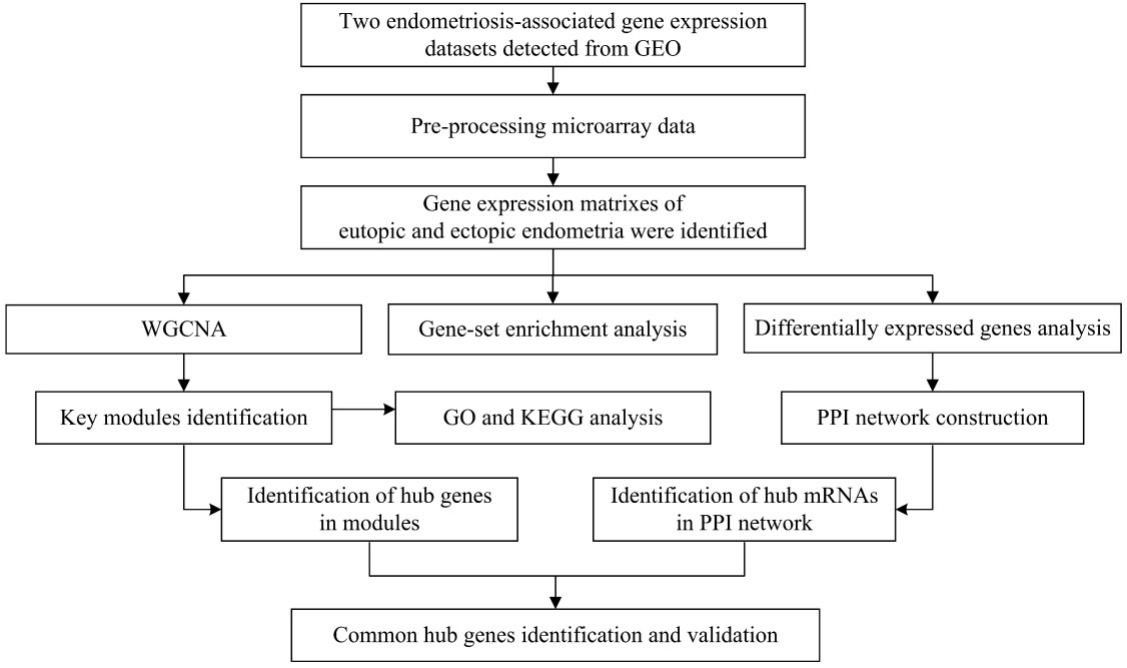
Flow diagram of strategy for data preparation, preprocessing, and analysis.

To illustrate the data preprocessing, analysis and validation, a schematic flow diagram of the study is presented in Figure 1.

### Data acquisition

Expression profiles of endometriosis-associated mRNAs in GSE7305, GSE120103, GSE7307 and GSE51981 were downloaded from Gene Expression Omnibus (GEO) database. The microarray datasets GSE7305 and GSE120103 with complete clinical information and same menstrual cycle were used as training sets to identify hub differentially expressed genes (DEGs) of endometriosis, GSE7307 and GSE51981 were used as test sets to validate our results, respectively. Dataset GSE7305 7 performed on the GPL570 platform (Affymetrix Human Genome U133 Plus 2.0 Array) was used to recognize hub DEGs in ovarian endometrioma, which includes 10 ovarian endometriomas from women with endometriosis (EC) and 10 normal endometria (Ctrl). Dataset GSE120103 8 performed on the GPL6480 platform (Agilent-014850 Whole Human Genome Microarray 4x44K G4112F) was applied to identify hub DEGs in eutopic endometrium, which includes 9 eutopic endometria from fertile women with endometriosis (EU) and 9 Ctrl. Dataset GSE7307 performed on the GPL570 platform (Affymetrix Human Genome U133 Plus 2.0 Array) was used to validate EC-associated hub DEGs, which includes 23 EC and 18 Ctrl. And Dataset GSE51981 performed on the GPL570 platform (Affymetrix Human Genome U133 Plus 2.0 Array) was used to validate EC-associated hub DEGs, which includes 38 EU and 71 Ctrl.

### Data preprocessing and differentially expressed genes (DEGs) identification

Limma (linear models for microarray data) package 9,10 in R software was utilized to correct the data background and identify the DEGs in EC vs Ctrl and EU vs Ctrl groups. Batch effect was removed using the limma package removeBatchEffect function. Benjamin and Hochberg method was used for multiple testing corrections 11. The false discovery rate (FDR) <0.05 and |log_2_ (Fold Change)| (|log_2_FC|) ≥1 was the cut-off criteria for screening DEGs.

### Gene-set enrichment analysis (GSEA)

To evaluate the molecular mechanisms of endometriosis, GSEA of the gene expression profiles of GSE7305 and GSE120103 was performed using the “ClusterProfiler” package in R (http://www.bioconductor.org/packages/release/bioc/html/clusterProfiler.html). The genes were listed based on their expression levels, and were further mapped to the annotated gene sets of c5 (Gene Ontology (GO) gene sets) and c2 (curated gene sets) in Molecular Signatures Database (MSigDB). Gene sets with P-value <0.05 and FDR <25% are considered as significant 12.

### Protein-protein interaction (PPI) network construction

The search tool for retrieval of interacting genes (STRING) database was used to identify the interactions among DEGs with the parameters of protein interaction score>0.4. Thereafter, the PPI network is constructed by Cytoscape. The potential hub DEGs were determined by Molecular Complex Detection (MCODE) plug-in (K-score>3) 13.

### Weighted gene co-expression network construction

We used the WGCNA R package to establish co-expression networks 14 for the genes in GSE7305 and GSE120103. The unqualified genes were screened out. A matrix of genes' similarity by Pearson's correlation analysis was created. Appropriate soft threshold power (β) was applied to strengthen this matrix to a scale-free co-expression network. For this purpose, we choose the lowest power (14 or 22) for which the scale-free topology fit index curve flattens out upon reaching a high value (above 0.8). Furthermore, the adjacency matrix was transformed into the topological overlap matrix (TOM). Genes with higher TOM values indicate higher connectivities in the network; that is, more adjacencies to other network-generated genes 15,16. Meanwhile, genes were clustered hierarchically by the TOM-based dissimilarity (1-TOM) measure. The highly correlated genes were assigned to the same module.

### Clinically significant module identification and function analysis

The correlation between modules and clinical traits was investigated by the module-trait relationship analysis of WGCNA. The modules that most relevant to the clinical traits could be identified. In this study, the endometriosis-associated blue and magenta modules were chosen for the subsequent analyses. Metascape was used to explore the function annotations (GO biological processes and Kyoto Encyclopedia of Genes and Genomes (KEGG) pathways) of these two modules. Terms with P-value<0.01, count≥3 and an enrichment factor>1.5 were considered statistically significant.

### Hub genes identification and verification

We analyzed the gene significance (GS, the correlation between the gene and a clinical phenotype of interest) and module membership (MM, the correlation between gene expression profile and module eigengene) of each gene in the clinically significant blue and magenta modules. The module eigengene is defined as the main component of the module's gene expression matrix. |MM|>0.6 and |GS|>0.8 were set as the threshold for screening candidate hub genes that strongly associated with EC or EU. In the end, the Venn diagram was performed to identify the common hub genes from PPI network analysis and WGCNA. In addition, GSE7307 and GSE51981 were used as validation data sets. “ggplot2” (Ito & Murphy, 2013) R package was applied to show relative expression of the identified hub genes in different comparison groups (EC vs Ctrl or EU vs Ctrl).

## Results

### Identification of differentially expressed genes

**Figure 2:**
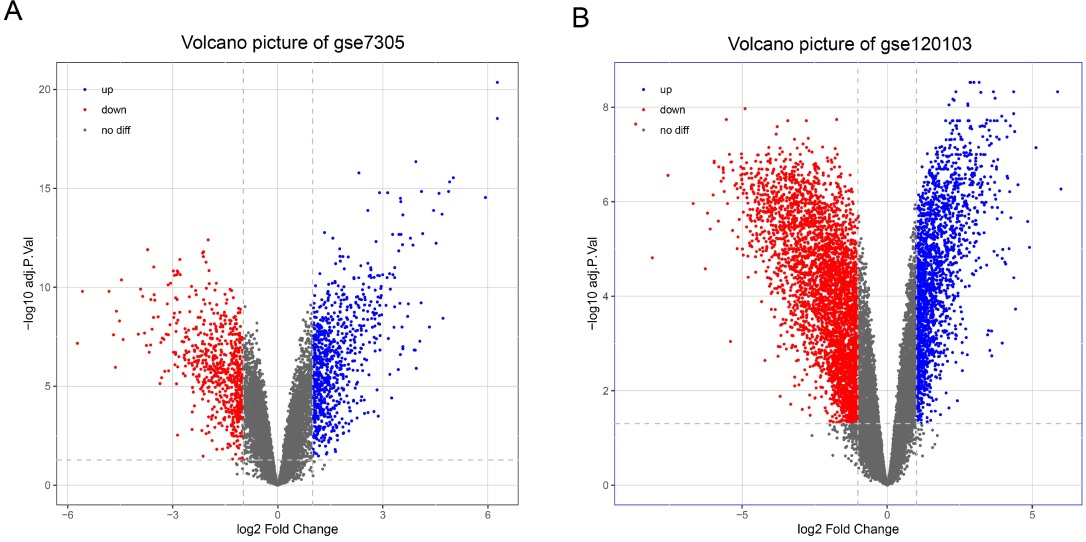
** Differentially expressed genes in endometriosis. (A)** Volcano map of differentially expressed genes in EC compared with Ctrl. **(B)** Volcano map of differentially expressed genes in EU compared with Ctrl. EC: ectopic endometrium from patient with endometriosis. EU: eutopic endometria from patient with endometriosis. Ctrl: endometrium from patient without endometriosis.

With the cut-off criteria (FDR<0.05 and |log_2_FC|>1), a total of 1487 DEGs (824 upregulated and 663 downregulated) were identified between the EC and Ctrl in the GSE7305 dataset, and a total of 5794 DEGs (1974 upregulated and 3820 downregulated) were identified between the EU and Ctrl in the GSE120103 dataset. Volcano plots show the variation of DEGs in the EC versus Ctrl (Figure 2A) and EU versus Ctrl (Figure 2B).

### Gene-set enrichment analysis

**Figure 3:**
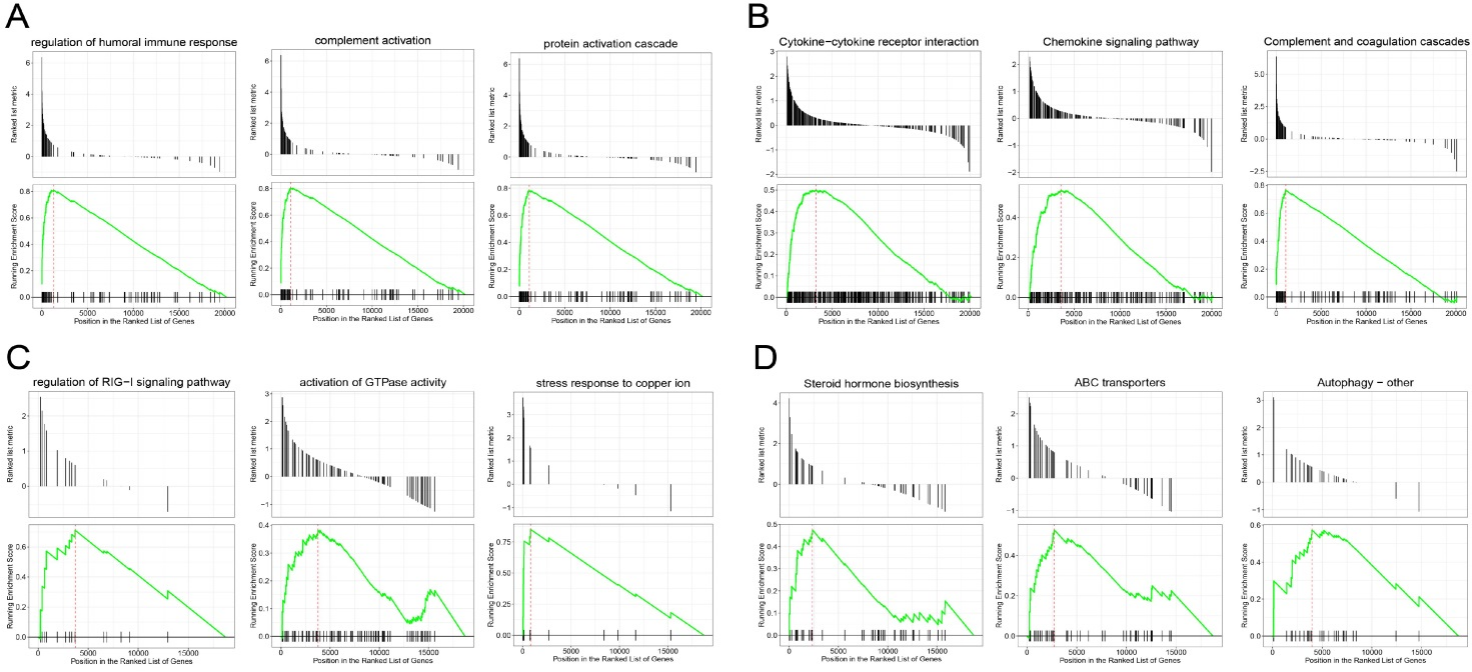
** Gene set enrichment analysis (GSEA) of genes in endometriosis.** GSEA-identified biological processes **(A)** and pathways **(B)** with significant enrichment in EC compared with Ctrl. GSEA-identified biological processes **(C)** and pathways **(D)** with significant enrichment in EU compared with Ctrl.

GSEA revealed the genes in the EC were mainly enriched in the immune response and immune cell trafficking, such as protein activation cascade, complement activation, regulation of humoral immune response, complement and coagulation cascades pathway and chemokine signaling pathway (Figure 3A-B), and the genes in the EU were mainly enriched in the stress response and steroid hormone biosynthesis, such as the stress response to copper ion, activation of GTP hydrolases (GTPases) activity, Human ATP-binding cassette (ABC) transporter dependent pathway and steroid hormone biosynthesis pathway (Figure 3C-D). We explored the functions of the two entities of endometriosis using genomic alteration profiles. These function annotations revealed the distinct roles of the EC and EU in the pathological process of endometriosis, which demonstrated the reliability of our results.

### Construction of protein-protein interaction network

**Figure 4:**
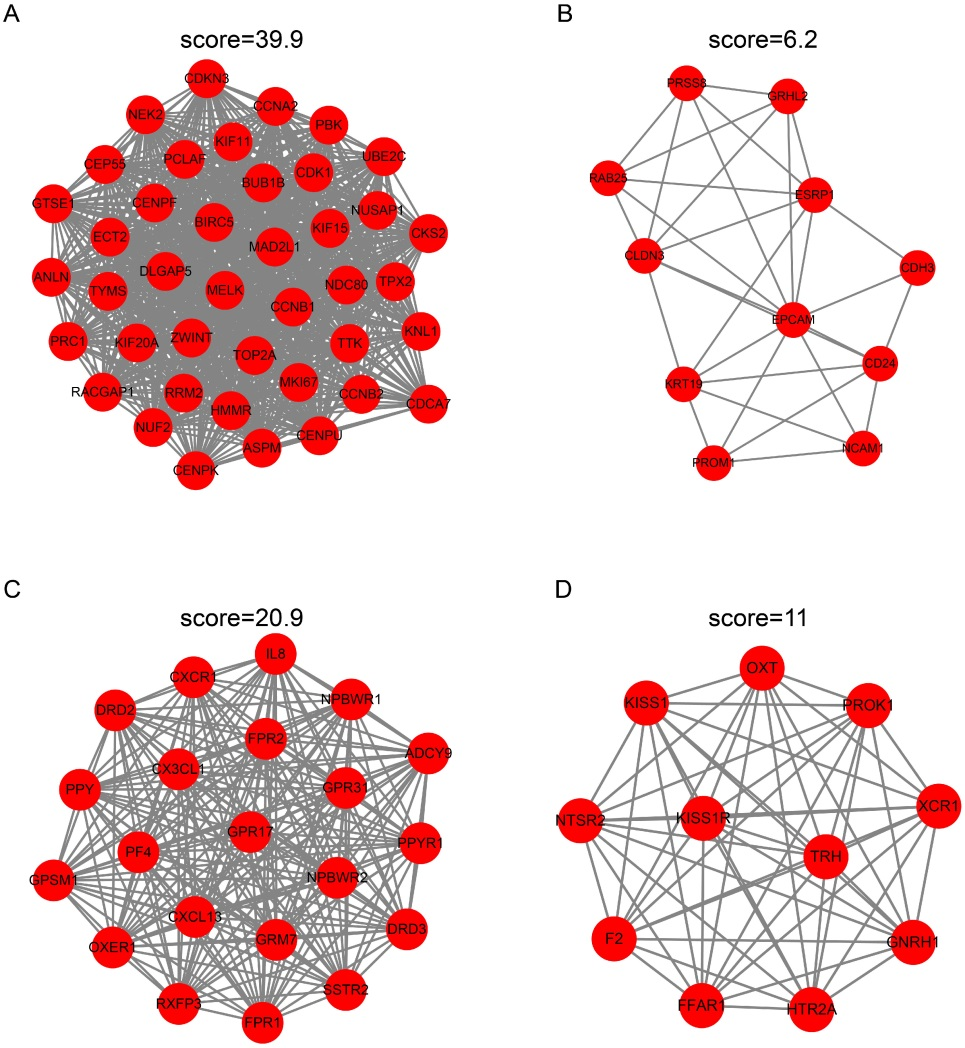
** Protein-protein interaction network cluster analysis.** The PPI network of differentially expressed genes in EC versus Ctrl or EU versus Ctrl was constructed using the STRING website. The MCODE clustering algorithm was applied to the network to identify the potential hub genes. Top two clusters in each group are shown in the figure. In the EC-associated PPI network, cluster 1 consists of 11 nodes and 31 edges **(A)** and cluster 2 consists of 11 nodes and 31 edges **(B)**. In the EU-associated PPI network, cluster 1 consists of 21 nodes and 209 edges **(C)** and cluster 2 consists of 11 nodes and 55 edges **(D)**. PPI: protein-protein interaction; STRING, search tool for retrieval of interacting genes/proteins.

To identify the candidate hub genes, the most differentially expressed 362 genes in the EC versus Ctrl and 1992 genes in the EU versus Ctrl were selected for the PPI network construction. The MCODE clustering algorithm was applied to analyze the PPI network. With a threshold of k-scores>3, seven clusters with 78 candidate hub genes in the EC and 21 clusters with 205 candidate hub genes in the EU were selected. Figure 4A-D depicts the top two clusters in the EC and EU.

### Construction of the weighted gene co-expression network and identification of clinically significant modules

**Figure 5:**
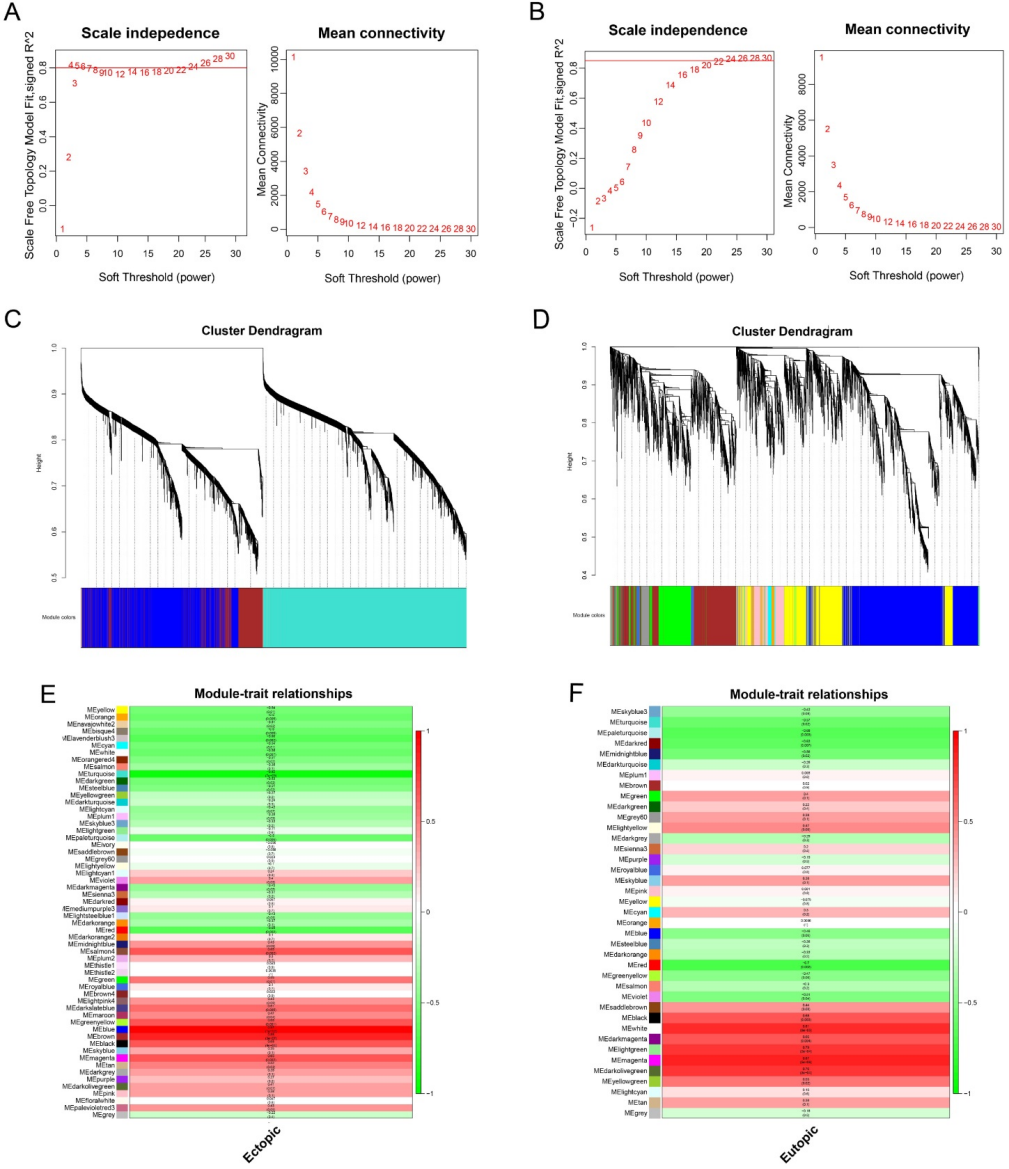
** Weighted gene co-expression network analysis of genes in endometriosis.** Analysis of the scale-free topology fit index and the mean connectivity for various soft-threshold powers (β) for the genes in the EC **(A)** and EU **(B)**. Dendrogram of all expressed genes in the EC **(C)** and EU **(D)** clustered based on a dissimilarity measure (1‐TOM). Determination of module-trait relationship of the EC **(E)** or EU **(F)** group in endometriosis and identification of the most clinically relevant modules; each row indicates a module eigengene (the first principal component of the gene expression matrix in a module), and each column represents a clinical trait. TOM: topological overlap matrix.

The values of β = 14 (scale free R^2^ = 0.80) and β = 22 (scale free R^2^ = 0.85) were selected as the soft-threshold powers to ensure scale-free networks using R package of “WGCNA” (Figure 5A-B). Genes with similar expression patterns were clustered into co-expression modules that were displayed in different colors. A total of 58 and 39 modules were identified (Figure 5C-D).

The relevance between each module and clinical information was shown in the module-trait relationship (Figure 5E-F). In this situation, we focused on the EC and EU-associated key modules. The blue module, containing 2036 genes, was most correlated with the EC (R=0.87, p=5×10^-6^). Meanwhile, the magenta module, containing 768 genes, was most correlated with the EU (R=0.97, p=1×10^-12^). Hence, the blue and magenta modules were clinically significant and used for the following analyses in this study.

### Function analysis of the most significant module

**Figure 6:**
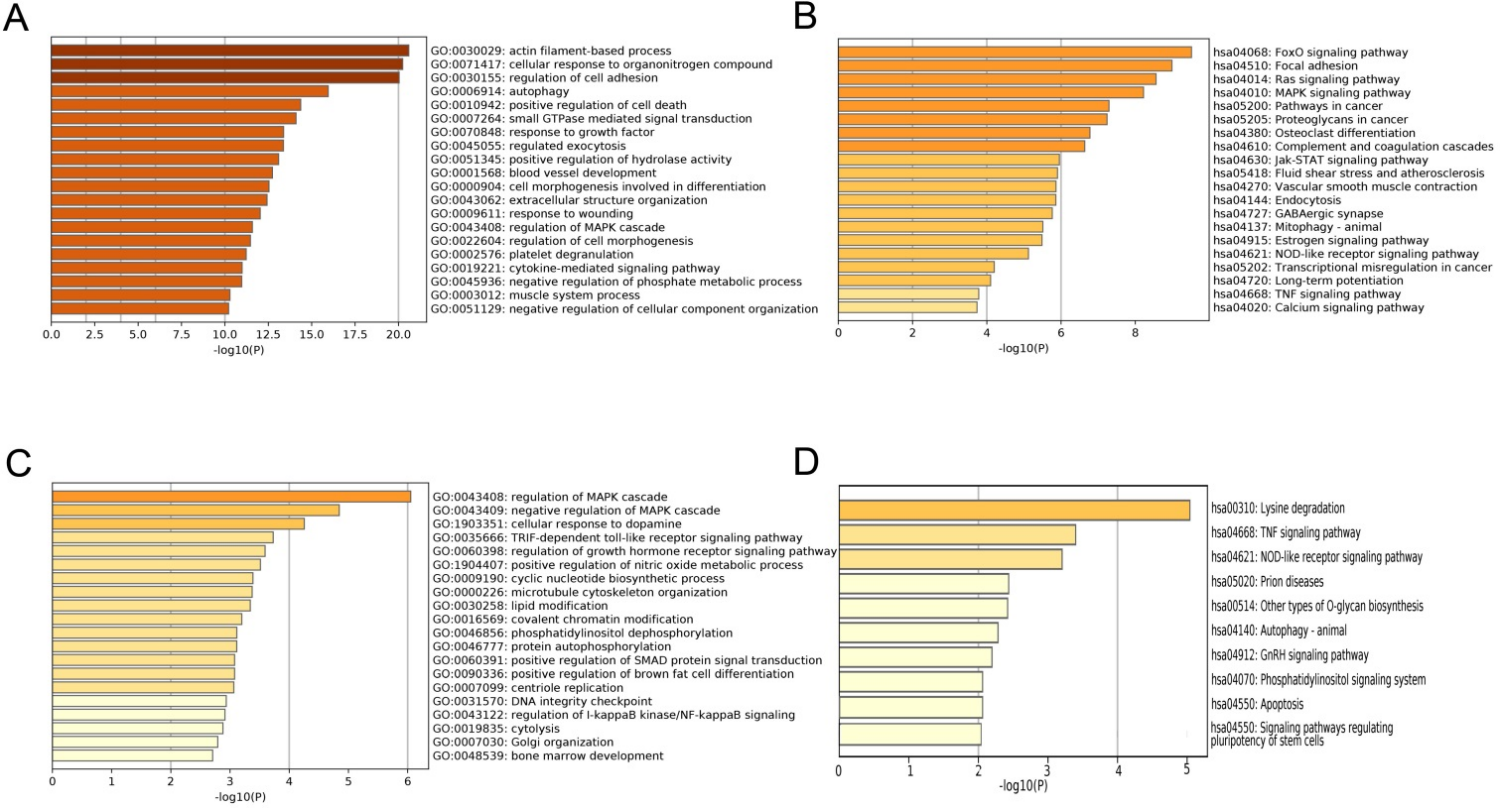
** Function annotations of clinically significant module.** The GO biological processes **(A)** and KEGG pathways **(B)** of genes in EC-associated blue module. The GO biological process **(C)** and KEGG pathway **(D)** of genes in EU-associated magenta module. GO: Gene Ontology; KEGG: Kyoto Encyclopedia of Genes and Genomes.

To explore the function mechanism of genes in the clinically significant modules, GO analysis and KEGG analysis were conducted. Function analysis revealed that the main biological processes and pathways of the blue module were regulation of cell adhesion, autophagy, FoxO signaling pathway and focal adhesion pathway (Figure 6A-B), and those of the magenta module were regulation of the mitogen-activated protein kinase (MAPK) cascade, regulation of the growth hormone receptor, the tumor necrosis factor (TNF) signaling pathway and NOD-like receptor signaling pathway (Figure 6C-D). These function annotations for the blue and magenta modules are listed in Table 1 and Table 2. The genes in the top EC-associated module mainly played roles in autophagy, focal adhesion and cancer, while those in the top EU-associated module were involved in creating an estrogen-rich and inflammatory microenvironment.

### Identification of hub genes

**Figure 7:**
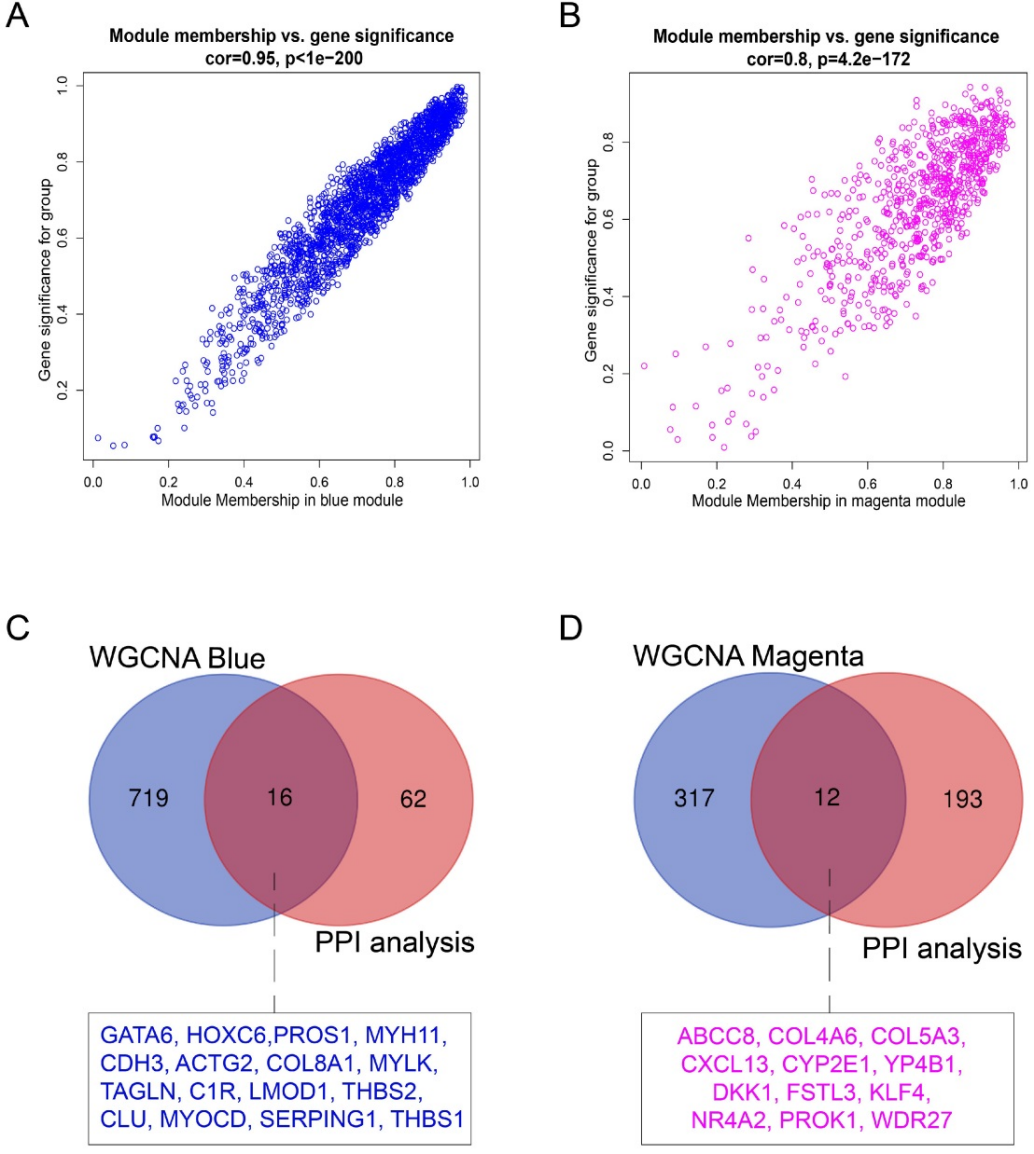
** Identification of hub genes in endometriosis. (A)** Scatterplot of module eigengenes related to EC in the blue co-expression module. **(B)** Scatterplot of module eigengenes related to EU in the magenta co-expression module. **(C)** Venn plot of common hub genes in blue module and PPI network. **(D)** Venn plot of common hub genes in magenta module and PPI network.

Hub genes in the co-expression network are characterized by high intramodular connectivity which is measured by the value of GS and MM. In Figure 7A and 7B, the scatterplots of GS (y-axis) vs. MM (x-axis) are shown in the blue (R=0.95, p<1×10^-200^) and magenta (R=0.8, p<4.2×10^-172^) modules. MM was highly correlated with GS in each module, which indicated that the hub genes in the co-expression modules were highly correlated with endometriosis. With the threshold of |MM| > 0.6 and |GS| > 0.8, using WGCNA, we identified 735 candidate hub genes in the blue module, and 329 candidate hub genes in the magenta module.

For the identification of endometriosis-associated hub genes, we compared the hub genes in the co-expression and PPI networks. We finally identified 16 overlapping hub genes in the blue module (Figure 7C) and 12 overlapping hub genes in the magenta module (Figure 7D). These 28 hub genes are listed in Table 3.

### Validation of hub genes

**Figure 8:**
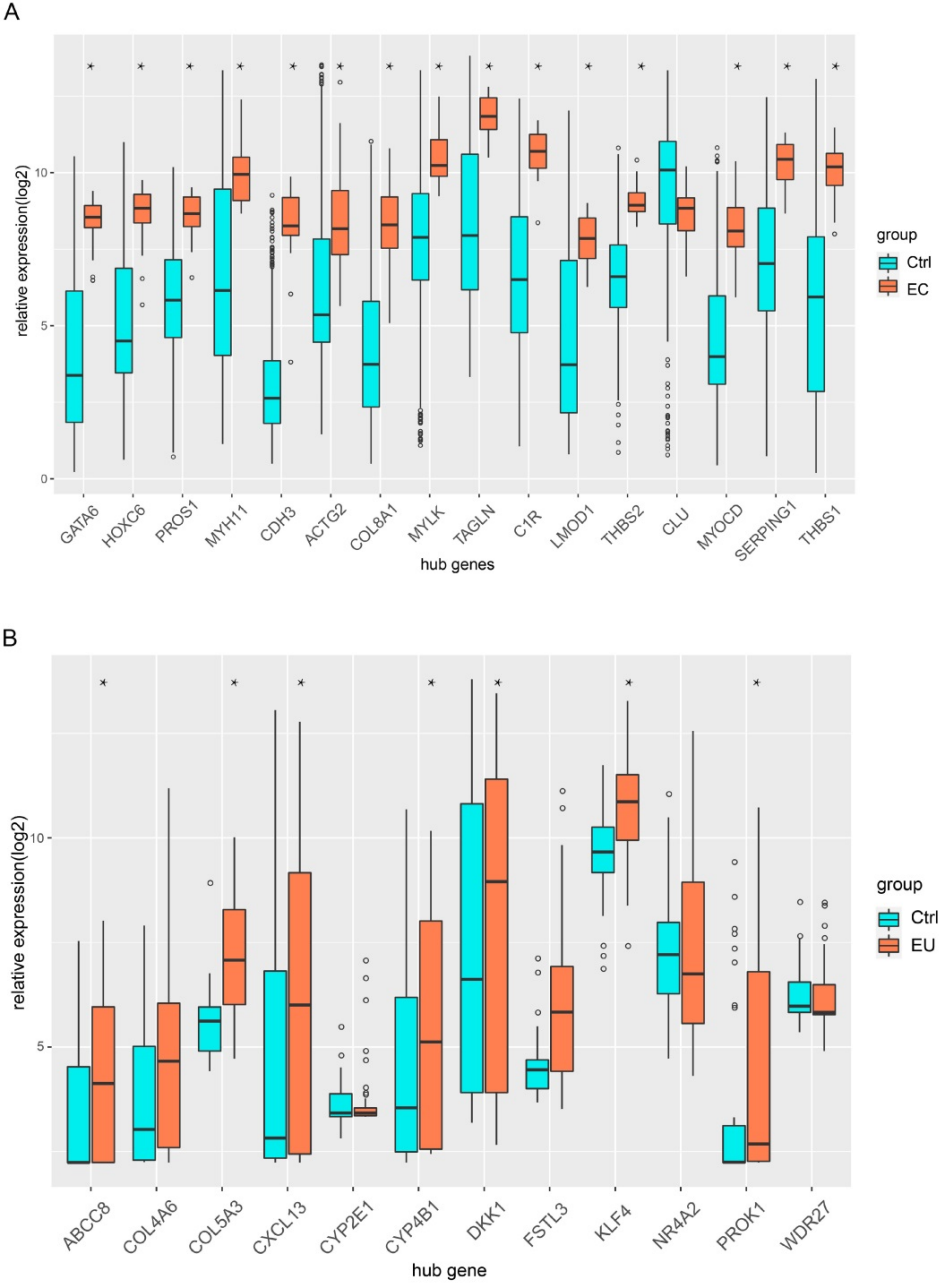
** Validation of hub genes in the independent GEO datasets. (A)** Boxplot shows the hub gene expression in EC and Ctrl. **(B)** Boxplot shows the hub gene expression in EU and Ctrl (*p< 0.05 versus Ctrl).

Independent datasets were used to identify the hub genes. We compared the expression of each hub gene in endometriosis. Fifteen hub genes were differentially expressed between the EC and Ctrl in GSE 7305 (Figure 8A), and seven hub genes were differentially expressed between the EU and Ctrl in GSE 51981 (Figure 8B). Boxplots were used to show the validation results (Figure 8A-B).

## Discussion

Endometriosis is a non-malignant gynecological disease whose pathogenesis is still unclear. The absence of biomarkers may contribute to the long delay between disease onset and diagnosis. Hence, it is imperative to identify novel molecular biomarkers that may enable early diagnosis and personalized treatment. For the first time, our study identified endometriosis-associated hub genes using WGCNA, which may hold important clues regarding the pathogenesis of endometriosis, provide valuable resources for the identification of endometriosis biomarkers and thus may improve the clinical management of this disease.

WGCNA can produce more robust results compared with other bioinformatics methods 17,18 because it constructs weighted co-expression networks based on the similarities of gene expression profiles and focuses on the correlation between the co-expressed modules and clinical traits. Hub genes are defined as the highly connected nodes that contribute to a phenotype or disease 19. Therefore, this method has been used to identify biologically relevant modules and biomarkers in different diseases 20. Endometriosis is a benign disease, although, similar to cancer, it has characteristics of being invasive and migratory. In our study, EC and EU-associated hub modules were identified. Function enrichment analyses showed that the genes in the blue and magenta modules had different roles and both were significantly associated with endometriosis, which demonstrated our analysis. For example, genes in the EC-associated blue module played roles in autophagy, focal adhesion (the initiation step for disease progression 21) and cancer, all of which were involved in the pathogenesis of endometriosis 22,23. Previous studies showed that S100A7 promoted the development of endometriosis by activating NF-kappaB signaling pathway 24. In our study, the genes in EU-associated magenta module played roles in the regulation of growth hormone receptor signaling pathway, NF-kappaB signaling and GnRH signaling pathway, which induced an estrogen-rich and inflammatory microenvironment involved in cell division, cell movement and survival in endometriosis 20,25-27. As a result, we assume that hormone receptor signaling or inflammatory microenvironment may promote the passing of EU through oviducts and migrating to the ovarian surfaces, and adhesion and autophagy correlated genes may induce the successful ectopic implantation of endometrium (EC) and formation of endometriotic lesions. Taken together, dysregulated genes in the EU may be responsible for the increased propensity of endometrial debris ectopic implantation and for early events that lead to the establishment of lesions. Dysregulated genes in the EC may contribute to the lesion formation and influence the progression of the disease.

To better understand the pathogenesis of endometriosis, WGCNA and PPI analyses were used to identify the EC and EU-associated hub genes. Some hub mRNAs, such as TAS2R3, TAS2R41, SERPING1, CASR, CCKAR, GPR55, HCRTR2, CRH, HTR5A, CFTR, and ENAM, were also key enriched genes in the GSEA. For instance, SERPING1 was involved in the complement and coagulation cascades, both NR4A2 and ABCC8 played important roles in ABC transporters, and CYP2E1 was involved in the pathway of steroid hormone biosynthesis. In addition, some identified hub genes of the EC (TAGLN, GATA6, CDH3, CLU, COL8A1, MYH11, MYOCD) and EU (CXCL13, DDK-1, KLF4, CYP2E1, CYP4B1 and PROK1) have been reported be associated with endometriosis. For example, TAGLN may be involved in cell invasion, migration, and differentiation in endometriosis 28. GATA6 is an essential gene in the activation of estrogen synthesis and may become a molecular marker in endometriotic lesions 29,30. Endometrial CXCL13 expression may play an important role in the pathophysiology of endometriosis 31. MiR-200b inhibited invasive growth in endometriosis by targeting KLF4 32. Dysregulated endometrial PROK1 expression may be correlated with the progesterone resistance of endometriosis 33. Most importantly, we discovered some novel and important genes, including HOXC6, PROS1, SERPING1, MYLK, ACTG2 and THBS2 in the ectopic endometrium, and NR4A2, ABCC8, COL4A6, COL5A3, FSTL3 and WDR27 in the eutopic endometrium. For example, HOXC6 was found to regulate the response to hormonal signals, and the overexpression of FSTL3 significantly improved angiogenesis and neovascularization in the induced pluripotent stem cells 34. These hub genes may provide new mechanisms for endometriosis and will be investigated in the future.

Our study identified multi-molecule biomarkers in endometriosis. However, some patients of the validation datasets had incomplete clinical information, which affected further data exploration. The identified genes will be further validated by clinical specimens and *in vitro* experiments for their application in endometriosis.

## Conclusion

Our study for the first time analyzed the gene expression files of the eutopic and ectopic endometrium in women with endometriosis using WGCNA, explored the distinct functions of the eutopic and ectopic endometrium, and identified co-expression modules and potential biomarkers for endometriosis. Our study may improve the understanding of the pathogenesis of endometriosis and provide references for endometriosis-associated biomarkers and therapeutic targets.

## Figures and Tables

**Table 1:** The GO and KEGG Pathway analysis of genes in the blue module

Category	Term	Description	Log(p-value)
GO Biological Processes	GO:0030029	actin filament-based process	-20.593297
GO Biological Processes	GO:0071417	cellular response to organonitrogen compound	-20.2406423
GO Biological Processes	GO:0030155	regulation of cell adhesion	-20.0365408
GO Biological Processes	GO:0006914	autophagy	-15.9477686
GO Biological Processes	GO:0010942	positive regulation of cell death	-14.3658968
GO Biological Processes	GO:0007264	small GTPase mediated signal transduction	-14.0979829
GO Biological Processes	GO:0070848	response to growth factor	-13.3811234
GO Biological Processes	GO:0045055	regulated exocytosis	-13.372418
GO Biological Processes	GO:0051345	positive regulation of hydrolase activity	-13.0915765
GO Biological Processes	GO:0001568	blood vessel development	-12.7380519
GO Biological Processes	GO:0000904	cell morphogenesis involved in differentiation	-12.5393524
GO Biological Processes	GO:0043062	extracellular structure organization	-12.4268848
GO Biological Processes	GO:0009611	response to wounding	-12.0333641
GO Biological Processes	GO:0043408	regulation of MAPK cascade	-11.5789888
GO Biological Processes	GO:0022604	regulation of cell morphogenesis	-11.4691072
GO Biological Processes	GO:0002576	platelet degranulation	-11.2263844
GO Biological Processes	GO:0019221	cytokine-mediated signaling pathway	-10.9869128
GO Biological Processes	GO:0045936	negative regulation of phosphate metabolic process	-10.9676618
GO Biological Processes	GO:0003012	muscle system process	-10.2900458
GO Biological Processes	GO:0051129	negative regulation of cellular component organization	-10.2118548
KEGG Pathway	hsa04068	FoxO signaling pathway	-9.5
KEGG Pathway	hsa04510	Focal adhesion	-9
KEGG Pathway	hsa04014	Ras signaling pathway	-8.6
KEGG Pathway	hsa04010	MAPK signaling pathway	-8.2
KEGG Pathway	hsa05200	Pathways in cancer	-7.3
KEGG Pathway	hsa05205	Proteoglycans in cancer	-7.2
KEGG Pathway	hsa04380	Osteoclast differentiation	-6.8
KEGG Pathway	hsa04610	Complement and coagulation cascades	-6.6
KEGG Pathway	hsa04630	Jak-STAT signaling pathway	-6
KEGG Pathway	hsa05418	Fluid shear stress and atherosclerosis	-5.9
KEGG Pathway	hsa04270	Vascular smooth muscle contraction	-5.9
KEGG Pathway	hsa04144	Endocytosis	-5.9
KEGG Pathway	hsa04727	GABAergic synapse	-5.8
KEGG Pathway	hsa04137	Mitophagy - animal	-5.5
KEGG Pathway	hsa04915	Estrogen signaling pathway	-5.5
KEGG Pathway	hsa04621	NOD-like receptor signaling pathway	-5.1
KEGG Pathway	hsa05202	Transcriptional misregulation in cancer	-4.2
KEGG Pathway	hsa04720	Long-term potentiation	-4.1
KEGG Pathway	hsa04668	TNF signaling pathway	-3.8
KEGG Pathway	hsa04020	Calcium signaling pathway	-3.7

Note: GO Biological Processes: Gene ontology analysis of biological process. KEGG: Kyoto Encyclopedia of Genes.

**Table 2:** The GO and KEGG Pathway analysis of genes in the magenta module

Category	Term	Description	Log(p-value)
GO Biological Processes	GO:0043408	regulation of MAPK cascade	-6.05131099
GO Biological Processes	GO:0043409	negative regulation of MAPK cascade	-4.84419894
GO Biological Processes	GO:1903351	cellular response to dopamine	-4.25433626
GO Biological Processes	GO:0035666	TRIF-dependent toll-like receptor signaling pathway	-3.72907579
GO Biological Processes	GO:0060398	regulation of growth hormone receptor signaling pathway	-3.59181535
GO Biological Processes	GO:1904407	positive regulation of nitric oxide metabolic process	-3.51272839
GO Biological Processes	GO:0009190	cyclic nucleotide biosynthetic process	-3.38699864
GO Biological Processes	GO:0000226	microtubule cytoskeleton organization	-3.37279545
GO Biological Processes	GO:0030258	lipid modification	-3.34130208
GO Biological Processes	GO:0016569	covalent chromatin modification	-3.19790154
GO Biological Processes	GO:0046856	phosphatidylinositol dephosphorylation	-3.11446184
GO Biological Processes	GO:0046777	protein autophosphorylation	-3.11274876
GO Biological Processes	GO:0090336	positive regulation of brown fat cell differentiation	-3.07693529
GO Biological Processes	GO:0060391	positive regulation of SMAD protein signal transduction	-3.07693529
GO Biological Processes	GO:0007099	centriole replication	-3.06412444
GO Biological Processes	GO:0031570	DNA integrity checkpoint	-2.93592311
GO Biological Processes	GO:0019835	cytolysis	-2.87954652
GO Biological Processes	GO:0007030	Golgi organization	-2.79292724
GO Biological Processes	GO:0048539	bone marrow development	-2.684576
KEGG Pathway	hsa00310	Lysine degradation	-5
KEGG Pathway	hsa04668	TNF signaling pathway	-3.4
KEGG Pathway	hsa04621	NOD-like receptor signaling pathway	-3.2
KEGG Pathway	hsa05020	Prion diseases	-2.4
KEGG Pathway	hsa00514	Other types of O-glycan biosynthesis	-2.4
KEGG Pathway	hsa04140	Autophagy - animal	-2.3
KEGG Pathway	hsa04912	GnRH signaling pathway	-2.2
KEGG Pathway	hsa04070	Phosphatidylinositol signaling system	-2.1
KEGG Pathway	hsa04210	Apoptosis	-2.1
KEGG Pathway	hsa04550	Signaling pathways regulating pluripotency of stem cells	-2

**Table 3:** The common hub genes of WGCNA and PPI analysis in the blue and magenta modules

Gene Symbol	Gene Description	Module	WGCNA				PPI Analysis	Limma Analysis		
			GS	GS P value	MM	MM, P value	K Score	Log2FC	FDR	Up or Down
ACTG2	actin, gamma 2, smooth muscle, enteric	blue	0.922139646	7.51E-09	0.923366176	6.54E-09	5.667	3.548158459	1.02E-08	up
C1R	complement C1r subcomponent	blue	0.897984733	7.81E-08	0.96174151	1.45E-11	4	2.208552292	1.14E-07	up
CDH3	cadherin 3	blue	0.931420936	2.48E-09	0.971836642	9.56E-13	6.2	2.775338929	3.97E-09	up
CLU	clusterin	blue	0.863575842	9.38E-07	0.878310934	3.54E-07	4	2.044619742	1.29E-06	up
COL8A1	collagen type VIII alpha 1 chain	blue	0.92193141	7.69E-09	0.941172973	6.47E-10	3	4.329881143	1.01E-08	Up
GATA6	GATA binding protein 6	blue	0.98763453	6.14E-16	0.972736196	7.16E-13	5.667	4.878194698	1.42E-15	up
HOXC6	homeobox C6	blue	0.944317318	3.99E-10	0.90875176	2.98E-08	4.8	3.487499137	6.36E-10	up
LMOD1	leiomodin 1	blue	0.880126801	3.12E-07	0.947799849	2.26E-10	5.667	2.476035697	4.14E-07	up
MYH11	myosin heavy chain 11	blue	0.933072838	2.00E-09	0.975751992	2.52E-13	5.667	3.708376486	2.96E-09	Up
MYLK	myosin light chain kinase	blue	0.914954646	1.62E-08	0.94723591	2.48E-10	5.667	2.125367821	2.54E-08	up
MYOCD	myocardin	blue	0.859995861	1.17E-06	0.929712545	3.08E-09	5.667	3.441041169	1.43E-06	up
PROS1	protein S (alpha)	blue	0.934607399	1.64E-09	0.984275338	5.27E-15	4	2.899254277	2.67E-09	up
SERPING1	serpin family G member 1	blue	0.859067114	1.23E-06	0.937390849	1.12E-09	4	2.424791429	1.61E-06	up
TAGLN	transgelin	blue	0.909061849	2.89E-08	0.959903156	2.20E-11	5.667	2.815676431	3.97E-08	up
THBS1	thrombospondin 1	blue	0.764480323	8.68E-05	0.84490284	2.77E-06	4	2.047575043	0.000118406	up
THBS2	thrombospondin 2	blue	0.870101501	6.18E-07	0.92128971	8.25E-09	3	2.397111323	8.17E-07	up
ABCC8	ATP binding cassette subfamily C member 8	magenta	0.726050115	0.000966995	0.922624053	1.35214E-07	3	2.368106299	0.000000474	Up
COL4A6	collagen type IV alpha 6 chain	magenta	0.831303264	3.54879E-05	0.873736048	4.59798E-06	4.71	3.592049292	5.82E-08	up
COL5A3	collagen type V alpha 3 chain	magenta	0.864722139	7.50448E-06	0.96496314	4.02139E-10	4.71	2.690812909	6.65E-08	up
CXCL13	C-X-C motif chemokine ligand 13	magenta	0.676038594	2.89E-03	0.808787134	8.47082E-05	20.9	2.692421845	0.00000669	up
CYP2E1	cytochrome P450 family 2 subfamily E member 1	magenta	0.813393515	7.1566E-05	0.937472578	2.85784E-08	4.5	2.3219305	4.78E-08	up
CYP4B1	cytochrome P450 family 4 subfamily B member 1	magenta	0.736111943	0.000754364	0.858320627	1.04106E-05	4.5	3.0752333	0.000000839	up
DKK1	dickkopf WNT signaling pathway inhibitor 1	magenta	0.697058489	0.001871854	0.815860925	6.53E-05	4.71	3.533265993	0.000000546	up
FSTL3	follistatin like 3	magenta	0.76382863	0.000358509	0.894831724	1.24E-06	5.067	2.997977144	0.000000244	up
KLF4	Kruppel like factor 4	magenta	0.725261009	0.000985574	0.884368596	2.45E-06	4.71	3.223838581	9.88E-08	up
NR4A2	nuclear receptor subfamily 4 group A member 2	magenta	0.800101777	0.000115047	0.864686086	7.52E-06	4.71	3.44866934	4.88E-08	up
PROK1	prokineticin 1	magenta	0.830706844	3.6373E-05	0.807977649	8.72E-05	11	4.082022638	1.93E-08	up
WDR27	WD repeat domain 27	magenta	0.790440633	0.000159037	0.950669894	5.02E-09	3	2.232448342	9.99E-08	up

WGCNA: weight gene co-expression network analysis, PPI: protein-protein interaction, GS: gene significance, MM: module membership, log_2_FC: log2 (Fold-Change) values of differentially expressed genes.

## Abbreviations

WGCNA: Weighted gene co-expression network analysis
GEO: gene expression omnibus
DEGs: Differentially expressed genes
PPI: Protein-protein interaction
Limma: linear models for microarray data
MCODE: Molecular Complex Detection
TOM: Topological overlap matrix
GO: Gene Ontology
KEGG: Kyoto Encyclopedia of Genes and Genomes
GSEA: Gene-set enrichment analysis
GS: gene significance
MM: module membership
STRING: The search tool for retrieval of interacting genes
